# Antibiotic Self-Prescribing Trends, Experiences and Attitudes in Upper Respiratory Tract Infection among Pharmacy and Non-Pharmacy Students: A Study from Lahore

**DOI:** 10.1371/journal.pone.0149929

**Published:** 2016-02-26

**Authors:** Zikria Saleem, Hamid Saeed, Mobasher Ahmad, Mahrukh Yousaf, Hafsa Binte Hassan, Ayesha Javed, Nida Anees, Sonu Maharjan

**Affiliations:** Clinical Pharmacy Section, University College of Pharmacy, University of the Punjab, AllamaIqbal Campus, 54000, Lahore, Pakistan; Centers for Disease Control and Prevention, UNITED STATES

## Abstract

Pharmacists are the custodians of drugs; hence their education, training, behaviors and experiences would affect the future use of drugs at community and hospital pharmacies. Therefore, we aimed at evaluating the self-prescribing antibiotic trends, knowledge and attitudes among pharmacy and non-pharmacy students. We found that pharmacy students had higher risks of experiencing URIs related symptoms such as cough (RR; 1.7, *p* = 0.002), allergy (RR; 2.07, *p* = 0.03) and running nose (RR; 3.17, *p*<0.005), compared to non-pharmacy students -resulting in higher probabilities of selecting cough syrups (OR; 2.3, *p*<0.005), anti-histamines (OR; 1.8, *p* = 0.036) and anti-inflammatory/anti-pyretic (OR; 2.4, *p*<0.005) drugs. Likewise, bachelor’s degree pupils (OR; 2, *p* = 0.045), urban area residents (OR; 2.44; *p* = 0.002) and pharmacy students (OR; 2.9, *p*<0.005) exhibited higher propensities of antibiotic self-use–notable classes include, b-lactams (45.9%) followed by macrolides (26.5%) and augmentin (28.94%), respectively. Surprisingly, pharmacy and non-pharmacy students had higher odds of using antibiotics in common cold (OR; 3.2, *p*<0.005) and pain (OR; 2.37, *p* = 0.015), respectively. Unlike non-pharmacy students, pharmacy students were likely to select alternative therapy, such as Joshanda (OR; 2.22, *p* = 0.011) and were well acquainted with antibiotic hazards, with 77% reduction in risk of antibiotics re-use. In conclusion, university students exhibited antibiotic self-prescribing trends in conditions that does not warrant their use, thus are irrational users. The pharmacy education confers very little benefit to rational self-prescribing practices among students, while non-pharmacy students are more vulnerable to repeated antibiotic usage. Thus, the educational and training modules should be designed for university students to disseminate targeted information regarding the potential hazards of antibiotic self-use and importance of consultation with qualified and registered medical doctor/pharmacist before starting with antibiotics.

## Introduction

Upper respiratory tract infections (URIs), the common acute infections, among others, are the most reported reasons of patient’s visits to the clinic [1]. Despite its mild features, short duration and self-limiting nature, URIs are among the leading causes of students absenteeism [2]. There is increasing evidence that antibiotics are often used inappropriately for URIs, since in majority of URIs, having viral cause, antibiotics confer little or no clinical benefit [3]. Thus, overuse of antibiotics can add not only to un-necessary drug therapy cost but also expose its user to un-wanted adverse effects[4,5]. Moreover, irrational use of antibiotics contributes majorly towards antibiotic resistance–more frequently observed in developing countries compared to developed world [6,7]. In developing countries, among other reasons, self-medication of antibiotics is a leading cause of increased antimicrobial resistance burden[8].

Several factors can contribute towards self-antibiotics use, such as financial status, medical knowledge, religious and cultural beliefs, education and uncertain diagnosis [9,10]. Surprisingly enough, data from previous studies suggested that self-medication trend is more prevalent in educated class in comparison to un-educated population[11,12]. Similarly, medical knowledge has also been shown to effect the self-medication practices based on two propositions, i-e., proper and improper knowledge of medicines, both impacting self-care orientation and can affect consumer’s attitude towards the consumption of medicines[13,14]. More strikingly, self-medication practice at an early age, i-e., university students, means that the habit could lead to a protracted use of medicines sans legitimate prescriptions, commonly generated upon the diagnosis of a medical condition. Moreover, students with URIs and living in hostels experience frequent infection episodes, possibly because of contagious nature of the infection and frequent traffic of infection carriers from other cities, transmissible via social gathering, combined study sessions and shared daily use facilities. Furthermore, sharing of disease and drug related knowledge and experiences resulted in treatment decisions adaptable and authenticated through convincing social gatherings that can promote self-medication practices.

Among University students, irrational use of antibiotics has been a common problem and is supported by several reports[15,16]. In this regard, studies have shown that University pharmacy students acquiring drugs knowledge, i-e., pharmacists, have the tendency to resort towards self-medication[11,17]. A pharmacist undergoes extensive training from drug manufacturing to its end use, thus their beliefs, habits and behaviors towards antibiotic use can have far reaching repercussions on the future use of antibiotics in a society, specifically in URIs. Seemingly, the continuing self-medication habits, knowledge of drugs, drug preferences and attitude of a pharmacist, custodian of drugs, would determiner future antibiotic consumption not only among pharmacists but also among community members interacting them. Therefore, it is pertinent to study antibiotics use practices in pharmacy and non-pharmacy graduates to identify the effect of pharmacy education on antibiotic utilization trends affecting future use. Thus, the study was aimed at evaluating self-antibiotic usage among pharmacy and non-pharmacy students of public and private universities of Lahore, Pakistan, in order to develop educational and training modules for university students to impress upon the importance of rational antibiotic use–vital for changing perceptions and behaviors that promote excessive and irrational use of antibiotics.

## Methodology

### Ethical Approval

Ethical approval for the study was obtained from Ethical Committee of Clinical Research, University College of Pharmacy, University of the Punjab, reference number (ECCR/UCP/08/2015) and Hospital committee of ethics on human research.

### Study Design

A cross sectional study of 9 months duration was conducted to evaluate the use of antibiotics in URIs among pharmacy (P) and non-pharmacy (NP) university students of Lahore, Pakistan. A comprehensive self-administered questionnaire was designed, as per study objectives, to be filled by the students, followed by a short interview by trained project administrators. The study was conducted in three public and several private Universities, i-e., University college of Pharmacy, University of the Punjab, University of Veterinary and Animal Sciences (UVAS), Government College University (GCU) and private universities of Lahore.

### Study Population

A total of 380 University students were enrolled for the study. Both male and female students, enrolled in various degree programs, Bachelors, Master/Mphil, were randomly selected with in the University premises to fill in the self-administered questionnaire. Randomization was ensured by asking the field administrators to enroll students at University premises, i-e., outside the classrooms, hostels and cafeteria, potential student’s gatherings, irrespective of study year, convenience and degree program. Non-pharmacy students were enrolled from various departments of the same Universities including History, Environmental Sciences, Microbiology, Commerce, Art and Design, Urdu, Economics and others (Fig A in S1 Text). Enrollees were segregated into two arms based on their enrollment in pharmacy and non-pharmacy degree programs. A total of 181 and 199 enrollments were confirmed in Pharmacy and non-pharmacy arms, respectively.

### Data Collection Procedure

Trained project field administrators, adept in data collection, visited various public and private universities of Lahore to enroll the students for data collection. A self-administered questionnaire was distributed among randomly selected respondents followed by a short interview to cross check and clarify any ambiguity in acquired information via filled questionnaire. The enrollees were asked to fill in the questionnaire on the basis of their last recent or past upper respiratory infections occurred not later than 6 months to reduce the recall bias. Informed consent was obtained from all enrollees before filling the self-administered questionnaire.

### Data Collection Tool

A comprehensive questionnaire, catering all the necessary project objectives was employed to estimate the frequency of irrational use of antibiotics for upper respiratory tract infections and to recognize the most widely used self-administered antibiotics in upper respiratory tract infections. The questionnaire consisted of 32 questions and was sub-divided into four major portions: (a) basic demographics like gender, age, residence, qualification and occupation, (b) symptoms that compel the use of antibiotics, (c) symptomatic treatment options opted by the respondents, (d) finally, drug use was evaluated to determine patient and prescriber role in rationale use of antibiotics by asking antibiotic therapy related questions.

### Data analysis

Data obtained were analyzed using Statistical Process for Social Sciences (SPSS 21)program. Pearson correlation was applied between total symptoms appeared and total medications used. Chi-square test was applied to check the association of different drug usage parameters and variables for URTIs between study groups. Univariate analysis was done to estimate the odds ratio for the variables affecting symptomatic drug therapy and antibiotic use. For ‘YES’ and ‘NO’ questions odds ratio was estimated using cross-tabs. An alpha value of ≤ 0.05 was considered statistically significant.

## Results

### Population’s Basic Demographics

**Fig 1:**
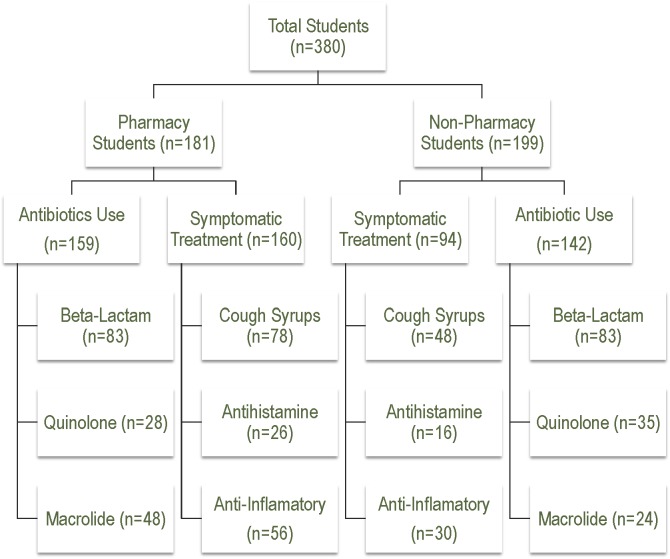
Flow Sheet for Drug Usage Among University Students.

The basic demographics of study population and flow sheet of drug usage overview among University students are summarized in Table 1 and Fig 1, respectively. The average age of the all the students was 21 years, ranging from 17 to 35years (21.8±1.7 pharmacy vs. 20.7±2.5 non-pharmacy, *p*<0.005). The frequency of female respondents was 74.73% compared to male respondents, 25.26%. Most of the respondents either male or female were single (93.22%), unemployed (90.14%)and belong to urban areas (86.23%). As shown in Table 1, majority of the students were enrolled in bachelor’s program (P; 89%, NP; 81.9%) in comparison to students enrolled in master/Mphil degree program (P; 11%, NP; 16.1%).

**Table 1:** Patient’s Basic Demographics.

Characteristics	Pharmacy Students (n = 181)	Non-pharmacy Students (n = 199)	Total (n = 380)
Age*			
	21.8±1.7	20.7±2.5	21.2±2.2
**Gender**			
Male	35(19.33%)	61 (30.65%)	96 (25.26%)
Female	146(80.66%)	138 (69.3%)	284 (74.73%)
**Residence**			
Rural	24 (13.3%)	49 (24.6%)	73 (19.2%)
Urban	157 (86.7%)	150 (75.4%)	307 (81.8%)
**Qualification**			
Bachelors	161 (89.0%)	163 (81.9%)	324 (85.3%)
Masters	20 (11.0%)	36 (18.1%)	56 (14.7%)
**Occupation**			
Unemployed	153(84.5%)	167 (83.9%)	320 (84.2%)
Self-employed	28 (15.5%)	32 (16.1%)	60 (15.8%)

* *Average age with standard deviation*

### Frequency and Risks of Symptoms Affecting Drug Choices and their Use

The frequency and relative risks of symptoms related to URTIs experienced by both pharmacy and non-pharmacy students are summarized in Table 2. In both groups, pharmacy (P) and non-pharmacy (NP), the most commonly observed symptoms include, cough (P; 51.93%, NP; 30.15%), headache (P; 39.77%, NP: 38.19%), cold (P; 23.75%, NP; 26.63%), fever (P; 32.59%, NP; 25.12%), running nose (P; 33.14%, NP; 10.05%)and sore throat (P; 29.83%, NP; 18.09%). Nonetheless, pharmacy students compared to non-pharmacy students demonstrated higher risk of developing symptoms encouraging drug use, such as cough (RR; 1.7, *p* = 0.002), allergy (RR; 2.07, *p* = 0.03) and running nose (RR; 3.17, *p*<0.005) (Table 2). All other symptoms reported in Table 2, their frequency and relative risks, were not significantly different between pharmacy and non-pharmacy students.

**Table 2:** Frequency and Relative Risk of Symptoms Compelling Drug Use.

Symptoms	Pharmacy students (n = 181)	Non pharmacy students (n = 199)	RR	*p*—value
**Cough**	94 (51.93%)	60 (30.15%)	1.72	0.002**
**Cold**	43 (23.75%)	53 (26.63%)	-	ns
**Fever**	59 (32.59%)	50 (25.12%)	-	ns
**Allergy**	49 (27.07%)	26 (13.06%)	2.07	0.039*
**Running nose**	60 (33.14%)	20 (10.05%)	3.17	<0.005**
**Watery secretions**	28 (15.46%)	14 (7.03%)	-	ns
**Mild aches**	12 (6.62%)	8 (4.02%)	-	ns
**Watery eyes**	37 (20.44%)	27 (13.56%)	-	ns
**Exhaustion**	22 (12.15%)	19 (9.54%)	-	ns
**Headache**	72 (39.77%)	76 (38.19%)	-	ns
**Generalized tiredness**	37 (20.44%)	32 (16.08%)	-	ns
**Chills**	11 (6.07%)	8 (4.02%)	-	ns
**Red and swollen mucous membranes**	10 (5.52%)	5 (2.51%)	-	ns
**Sore throat**	54 (29.83%)	36 (18.09%)	-	ns
**Hoarseness**	5 (2.76%)	8 (4.02%)	-	ns
**Swollen lymph nodes**	6 (3.31%)	10 (5.02%)	-	ns
**Pain on swallowing**	22 (12.15%)	17 (8.54%)	-	Ns

*p-value ≤ 0.05

** p-value ≤ 0.005

ns = non-significant; **RR**; relative risk

Next we examined different variables that can affect the likelihood of drugs being used for symptomatic treatment. Interestingly enough, compared to non-pharmacy students, pharmacy students exhibited higher probabilities of selecting cough syrups (OR; 2.3, *p*<0.005), anti-histamines (OR; 1.8, *p* = 0.036) and anti-inflammatory/anti-pyretic (OR; 2.4, *p*<0.005) drugs. Moreover, an upward trend was observed in students hailing from urban areas (urban; 12.1%, rural; 6.8%), compared to students from rural areas regarding the use of cough syrups, antihistamine and anti-inflammatory/anti-pyretic drugs as symptom relievers (Table 3). While other variables, such as gender, employment status and degree programs no significant differences were observed regarding the use of drugs in symptomatic treatment of URIs (Table 3).

**Table 3:** Analysis of Variables Affecting Drug Use in Symptomatic Treatment of URIs.

Symptomatic Treatment Options and Factors Affecting their Use				
Symptomatic Treatment Options	Variables	Respondents (%)	X^2^-test (*p*-value)	OR
**Cough Syrups**	Female	99 (34.9%)	ns	-
	Male	27 (28.1%)		
	Rural	19 (26.0%)	ns	1.521
	Urban	107 (34.09%)		
	Bachelors	113 (34.9%)	ns	-
	Masters	13 (23.2%)		
	Self-employed	21 (35.0%)	ns	-
	Unemployed	105 (32.8%)		
	Pharmacy	78 (43.1%)	< 0.005**	2.3
	Non-Pharmacy	48 (24.1%)		
**Antihistamine**	Female	35 (12.3%)	ns	-
	Male	7 (7.3%)		
	Rural	5 (6.8%)	ns	1.864
	Urban	37 (12.1%)		
	Bachelors	37 (11.4%)	ns	-
	Masters	5 (8.9%)		
	Self-employed	6 (10.0%)	ns	-
	Unemployed	36 (11.3%)		
	Pharmacy	26 (14.4%)	0.036*	1.8
	Non-Pharmacy	16 (8.0%)		
**Anti-inflammatory/Anti-pyretic**	Female	67 (23.6%)	ns	-
	Male	19 (19.8%)		
	Rural	13 (17.8%)	ns	1.440
	Urban	73 (23.8%)		
	Bachelors	73 (22.5%)	ns	-
	Masters	13 (23.2%)		
	Self-employed	13 (21.7%)	ns	-
	Unemployed	73 (22.8%)		
	Pharmacy	56 (30.9%)	< 0.005**	2.4
	Non-Pharmacy	30 (15.1%)		

*p-value ≤ 0.05

** p-value ≤ 0.005

ns = non-significant. **OR;** odds ratio

### Effect of Demographic Variables on Antibiotic Use and Related Untoward Effects Among University Students

Next obvious question was to assess the antibiotic use among university students, commonly used brands and their pertinent side effects, as summarized in Table 4 and in Table A and B in S1 Text. When the effect of demographic variables was analyzed regarding antibiotic use, bachelor’s students (OR; 2, *p* = 0.045) compared to master students, residents of urban areas(OR; 2.44; *p* = 0.002) compared to rural occupants (Table 4) demonstrated higher odds of using antibiotics. Similarly, pharmacy education contributed significantly in increasing the prospects of antibiotic use (OR; 2.9, *p*<0.005) in comparison to non-pharmacy education (Table 4). Moreover, among antibiotic brands, self-employed (OR; 1.9, *p* = 0.036) and pharmacy students (OR; 2.5, *p*<0.005) exhibited higher propensity of using macrolides (Table 4). Additionally, β-lactams were more frequently used antibiotic (P; 45.9%, NP; 41.7%) compared to macrolides (P; 26.5%, NP; 12.1%) and are more likely to be used by bachelors (OR; 2.4, *p* = 0.004) and un-employed students (OR; 1.834, *p* = 0.027) and students from urban areas (OR; 1.757, *p* = 0.025) (Table 4). Interestingly, among the students, Augmentin (amoxicillin + clavulanic acid) was the most commonly used antibiotic brand for URIs related symptoms (28.94%) followed by Amoxil (20.35%) and Leflox (11.05%) (Table A in S1 Text).

**Table 4:** Analysis of Variables Affecting Antibiotic Use Among University Students.

Antibiotic Use and Factors Affecting their Use				
Drug = Usage	Variables	Number of Respondents (%)	X^2^-test (*p*-value)	OR
**Antibiotics**	Female	224 (80.2%)	ns	-
	Male	77 (78.9%)		
	Rural	48 (65.8%)	0.002**	2.440
	Urban	253 (82.4%)		
	Bachelors	262 (80.9%)	0.045*	2
	Masters	39 (69.6%)		
	Self-employed	49 (81.7%)	ns	-
	Unemployed	252 (78.8%)		
	Pharmacy	159 (87.8%)	<0.005**	2.9
	Non-Pharmacy	142 (71.4%)		
**Macrolide**	Female	53 (18.7%)	ns	-
	Male	19 (19.8%)		
	Rural	15 (20.5%)	ns	-
	Urban	57 (18.6%)		
	Masters	14 (25.0%)	ns	1.529
	Bachelors	58 (17.9%)		
	Self-employed	17 (28.3%)	0.036*	1.9
	Unemployed	55 (17.5%)		
	Pharmacy	48 (26.5%)	<0.005**	2.5
	Non-Pharmacy	24 (12.1%)		
**Beta-Lactam Antibiotics**	Female	124 (43.7%)	ns	-
	Male	42 (43.8%)		
	Urban	142 (46.3%)	0.025*	1.757
	Rural	24 (32.9%)		
	Bachelors	151 (46.6%)	0.004**	2.4
	Masters	15 (26.8%)		
	Self-employed	19 (31.7%)	0.027*	1.834
	Unemployed	147 (45.9%)		
	Pharmacy	83 (45.9%)	ns	-
	Non-Pharmacy	83 (41.7%)		
**Quinolones**	Female	47 (16.5%)	ns	-
	Male	16 (16.7%)		
	Rural	9 (12.3%)	ns	1.518
	Urban	54 (17.6%)		
	Bachelors	53 (16.4%)	ns	-
	Masters	10 (17.9%)		
	Self-employed	13 (21.7%)	0.166	0.670
	Unemployed	50 (15.6%)		
	Pharmacy	28 (15.5%)	ns	-
	Non-Pharmacy	35 (17.6%)		

*p-value ≤ 0.05

** p-value ≤ 0.005

ns = non-significant. **OR;** odds ratio

Next we assessed antibiotic use associated side effects in both the groups. List of commonly experienced side effects are shown in Table B in S1 Text. Data suggested that headache was the main side effect experienced by non-pharmacy students (37.68%)followed by laziness (24.12%) and sleeplessness (14.07%). On the other hand, most of the pharmacy students experienced laziness (28.96%), headache (25.17%) and fatigue (17.67%), as notable side-effects of antibiotic use.

### Knowledge, Attitudes, Clinical Experiences, Preferences and Pre-disposing Risks Promoting Drug Use Among University Students

Next obvious question was to assess the knowledge, attitudes, clinical experiences and preferences defining students drug choices and patterns in both the groups, i-e., pharmacy and non-pharmacy. When assessed for drug use patterns; knowledge and attitudes, only pharmacy students exhibited higher probabilities to consult general practitioner (OR; 3.7, *p*<0.005) and received medication orders from registered medical practitioner (OR; 2.5, *p* = 0.066) compared to non-pharmacy enrollees (Table 5).

**Table 5:** Drug Use Pattern; Clinical Experiences and Preferences in URIs.

Questions Regarding Clinic Knowledge & Experience	PHARMACY STUDENTS (N = 181)	NON-PHARMACY STUDENTS (N = 199)	X^2^-TEST *P*—VALUE	OR
**Have you undergone a lab test?**				
YES	19 (10.49%)	40 (20.10%)	ns	-
NO	162 (89.50%)	159 (79.89%)		
**Did your doctor prescribe any antibiotic?**				
YES	131 (72.37%)	135 (67.83%)	ns	-
NO	50 (27.62%)	64 (32.16%)		
**Did your doctor specify your medical condition?**				
YES	58 (32.04%)	86 (43.21%)	ns	-
NO	123 (67.95%)	113 (56.78%)		
**Preferred dosage form**				
Tablet/Capsule				
YES	151 (85.39%)	150 (83.33%)	ns	-
NO	30 (16.6%)	49 (24.6%)		
**S**yrup				
YES	26 (14.36%)	30 (15.07%)	ns	-
NO	155 (85.6%)	169 (84.92%)		
**Preferred consultation**				
**G**eneral practitioner				
YES	65 (35.9%)	26 (13.06%)	<0.005**	3.7
NO	116 (64.1%)	173 (86.93%)		
**C**onsultant				
YES	21 (11.6%)	39 20.41%)	ns	-
NO	160 (88.4%)	160 (80.04%)		
**P**rivate				
YES	75 (41.43%)	106 (53.26%)	ns	-
NO	106 (58.56%)	93 (46.73%)		
**P**ublic				
YES	16 (8.83%)	17 (8.54%)	ns	-
NO	165 (82.91%)	182 (91.45%)		
**Preferred medication order**				
**S**elf				
YES	19 (10.49%)	18 (9.04%)	ns	-
NO	162 (89.5%)	181 (90.95%)		
**RMP**				
YES	40 (22.09%)	20 (10.05%)	0.066	2.5
NO	141 (77.9%)	179 (89.94%)		
**R**elative				
YES	12 (6.62%)	25 (12.56%)	ns	-
NO	169 (93.37%)	174 (87.43%)		
**P**harmacist				
YES	8 (4.90%)	10 (5.40%)	ns	-
NO	173 (95.58%)	189 (94.97%)		

**p-value ≤ 0.005

ns = non-significant. **OR**; odds ratio, **RMP**; registered medical practitioner

Yet, majority of students, pharmacy and non-pharmacy did not go for a lab test before using antibiotics (P; 91.01% vs. NP; 82.98%). Surprisingly, most of the students claimed that antibiotics were started on doctor’s prescription (P; 73.59% vs. NP; 70.31%) but without specifying the medical condition (P; 64.41% vs. NP; 51.95%), yet upon cross questioning the claims proved to be invalid and turned out to be of several months (~ 6–9 months) old. Interestingly, upon further analysis, non-pharmacy students demonstrated higher propensity of reading the medication instructions (OR; 4, *p*<0.005), but still had higher probability of using antibiotics in pain (OR; 2.37, *p* = 0.015) compared to pharmacy students (Table 6). Surprisingly enough, pharmacy students demonstrated increased chances of using antibiotics in common cold (OR; 3.2, *p*<0.005),though not rationale, in comparison to non-pharmacy counter parts (Table 6). Strikingly, university students, pharmacy and non-pharmacy, preferentially self-medicate themselves in case of common cold (P; 39.77%, NP; 39.69%), mostly starting with antibiotics at home (P; 62.43%, NP; 51.75%) or as advised by doctor (P; 53.59%, NP; 49.74%), yet, strictly following the prescribed dose (P; 75.13%, NP; 61.30%)sans any botheration of a missing dose (P; 69.61%, NP; 65.82%). However, pharmacy students were more inclined towards herbal remedies such as Joshanda (OR; 2.22, *p* = 0.011) in comparison to non-pharmacy students (Table 6).

**Table 6:** Drug Utilization; Knowledge and Attitude Drifts in URIs.

Trends & Knowledge	PHARMACY (N = 181)	NON-PHARMACY (N = 199)	X^2^-TEST (*P*-VALUE)	OR
**Any alternative therapy**				
Joshanda	113 (62.43%)	85 (42.71%)	0.011*	2.22
Vitamin C	22 (12.15%)	30 (15.07%)	ns	-
**Preference in case of common cold?**				
Go to doctor	33 (18.96%)	46 (23.95%)	ns	-
Go to nearby pharmacy	9 (4.97%)	11 (5.52%)	ns	-
Self medicate yourself	72 (39.77%)	79 (39.69%)	ns	-
Home remedy	60 (33.14%)	56 (28.14%)	ns	-
**Did you read the instructions?**				
YES	60 (33.14%)	133 (66.83%)	<0.005**	4.06
NO	121 (66.85%)	66 (33.16%)		
**Do you understand instructions?**				
YES	155 (85.63%)	161 (84.29%)	ns	-
NO	26 (14.52%)	38 (20.76%)		
**How did you start antibiotic?**				
Without prescription	113 (62.43%)	103 (51.75%)	ns	-
With prescription	27 (14.91%)	40 (20.10%)	ns	-
With pharmacist’s advise	34 (18.78%)	40 (20.01%)	ns	-
**What was the purpose of antibiotic?**				
For fever	50 (27.62%)	65 (32.66%)	ns	-
For pain	38 (20.99%)	77 (38.69%)	0.015*	2.37
For malaise and fatigue	18 (9.94%)	13 (6.53%)	ns	-
For common cold	66 (36.46%)	30 (15.07%)	<0.005**	3.2
**How long did you use antibiotics?**				
Until finished	6 (3.31%)	10 (5.02%)	ns	-
Until symptoms relived	74 (40.88%)	76 (38.19%)	ns	-
As advised by the doctor	97 (53.59%)	99 (49.74%)	ns	-
**Did you follow the prescribed dose?**				
YES	136 (75.13%)	122 (61.30%)	ns	-
NO	34 (18.78%)	60 (30.15%)		
**What did you do if you miss any dose?**				
Twice dose next time	9 (4.97%)	10 (5.02%)	ns	-
Don’t bother	126 (69.61%)	131 (65.82%)	ns	-
Ask the doctor	38 (20.99%)	46 (23.11%)	ns	-

*p-value ≤ 0.05

** p-value ≤ 0.005

ns = non-significant. **OR**; odds ratio

Finally, factors that promote self-medication risks were examined among University students (Table 7). Data suggested that majority of students, pharmacy and non-pharmacy, answered ‘NO’ (P; 64.64%, NP; 66.83%) when asked about antibiotic self-prescription (Table 7). Similarly, in case symptoms persisted or experienced the same illness, more than 50%students opted to consult the doctors (persistent illness; P; 79.55%, NP; 66.33%, same illness again; P; 51.93%, NP; 55.77%), however, as expected, there were 19% more chances that pharmacy students considered antibiotics hazardous (RR; 1.2, *p* = 0.036) in comparison to non-pharmacy students (Table 7). Moreover, to avoid next attack, pharmacy students demonstrated 77% reduction in risk of using antibiotics (RR; 0.23, *p*<0.005) in comparison to non-pharmacy students, while pharmacy students demonstrated 2.04 times higher risk of taking no further action (RR; 2.04, *p*<0.005).

**Table 7:** Pre-disposing Factors that Promote Self-medication Risks.

Self-medication pre-disposing factors	Pharmacy (n = 181)	Non-pharmacy (n = 199)	*p*–value	RR
**Prior antibiotic self-prescribing experience**				
YES	64 (35.35%)	66 (33.16%)	ns	-
NO	117 (64.64%)	133 (66.83%)		
**What if symptoms persist?**				
Ask the doctor	144 (79.55%)	132 (66.33%)	ns	-
Ask the pharmacist	13 (7.18%)	25 (12.56%)	ns	-
Try home remedy	16 (8.83%)	20 (10.05%)	ns	-
Double the dose	7 (3.86%)	14 (7.03%)	ns	-
**Experienced the same illness again**				
Follow the old prescription	50 (27.62%)	43 (21.60%)	ns	-
Go to the doctor again	94 (51.93%)	111 (55.77%)	ns	-
Ask the pharmacist	14 (7.73%)	13 (6.53%)	ns	-
Try home remedy	22 (12.15%)	23 (11.55%)	ns	-
**Did you consider antibiotic dangerous?**				
YES	157 (86.74%)	144 (72.36%)	0.036*	1.2
NO	24 (13.25%)	55(29.64%)		
**In case of next attack**				
Symptomatic treatment	43 (23.75%)	50 (25.12%)	ns	-
Antibiotics	12 (6.62%)	58 (29.14%)	<0.005**	0.23
Alternativel THERAPY	28 (15.73%)	32 (16.75%)	ns	-
No action	95 (52.48%)	51 (25.62%)	<0.005**	2.04

*p-value ≤ 0.05

** p-value ≤ 0.005

ns = non-significant. **RR**; relative risk

## Discussion

Numerous studies have shown that the students studying medical and non-medical subjects and suffering from URIs develop self-medication habits during their University times that not only continue in their lives ahead but also more likely to promote such practices, despite custodians of rationale drug use[7,16,18]. In our study, we found that several variables, i-e., pharmacy education, URIs related symptoms, degree program, area of residence and employment status significantly increased the odds of using symptomatic treatments and self-prescribed antibiotics, though mindful about the hazards of antibiotic use. Moreover, pharmacy knowledge did not significantly oblige the students to rationalize and become conscientious about the drug choices and medication attitudes—evident by antibiotic consumption in common cold and not reading the instructions carefully. However, pharmacy students did consider alternative therapies, i-e.,Joshanda at the first instance to avoid antibiotic consumption, while non-pharmacy students consumed antibiotics in pain–a perfect example of antibiotic irrational use. Likewise, studies from Karachi reported the frequency of 31.2% and 27% of Joshanda use among children with common cold and acute respiratory infections, respectively, by the mothers[19,20].

Several lines of evidences suggested that symptoms related to respiratory tract infections (RTIs) are the frequent reasons of seeking health care services[21,22].Further studies suggested that in upper respiratory infections (URIs),general practitioners frequently prescribe antibiotics[23,24], yet half are irrational[25]. In this context, studies have shown that compared to general population, doctors and pharmacists are the vilest offenders of prescription drugs[11,18], as evident by rampant self-prescribing trends among doctors—56% among Norwegian[26], 53% among Indian[27], 25% among Brazilian[28] and also among the pharmacists [29]. Studies have also shown that these self-prescribing habits are acquired during the course of health professional’s education and training[17].Our data provide an empirical support to above mentioned studies that, in comparison to non-pharmacy students, pharmacy students were and more likely (OR; 2.9, *p*<0.005) to be the frequent self-prescribers of antibiotics (62%) and drugs used in symptomatic treatments. Thus, pharmacy students showed trivial prudence when it comes to the use of antibiotics, showing that antibiotic utilization trends are driven by social and cultural behaviors rather than knowledge per se. A similar trend has been observed for the antibiotic class, i-e., macrolides (OR; 2.5, p<0.005), nevertheless only 35.5% conceded to this practice. These findings were substantiated by another study demonstrating that medical students are more likely to use antibiotics compared to non-medical students[13]. Additionally, pharmacy students, compared to non-pharmacy students, exhibited higher likelihood of experiencing symptoms like, cough (P; 51.93%, NP; 30.15%), allergy (P; 27.07%, NP; 13.06%) and running nose (P; 33.14%, NP; 10.05%) that compel drug use, while other prevalent symptoms include headache, fever and cold that demonstrated almost equal frequencies among the students–the probable reason could be the ability of pharmacy students to perceive and feel the incommodious symptoms along with frequent combined study sessions and gatherings promoting transmission risks. Similar observations have been reported before in a study from Karachi, Pakistan, and India, where fever (41.1%), pain (40.1%) and headache were the most frequent symptoms enthralling self-medication[7,30]. Further analysis suggested that a low percentage of students, pharmacy and non-pharmacy, were taking antibiotics with prescription (≤ 20%), however, situation became more precarious when antibiotics were begun without a lab test (> 78%) and without comprehending a medical condition (> 55%), necessitating antibiotic use.

Moreover, this higher self-medication prevalence among students acquiring medical knowledge has been attributed to unbiased sources of information coupled with progressive knowledge gained about the drugs and disease[30,31].Yet, contrary to this notion, that acquiring medical knowledge would promote rational self-care practices, our data suggested that, compared to non-pharmacy students (15.07%),36.46% of pharmacy students utilize antibiotics in common cold -actually caused by a virus. Notwithstanding this fact of antibiotic usage in common cold, pharmacy students preferentially consulted general practitioners (P; 39.9%, NP; 13.06%) and received medication orders from registered medical practitioners (P; 22.09%, NP; 10.05%), though not practiced by many -signifying two major facts; a) pharmacy students were more aware of the risks and precautions associated with self-medication of antibiotics, thus are more cautious, b) presumably, medical practitioners were also irrational prescribers of antibiotics. However, these results cannot be generalized to a specific pharmacy-teaching year, since we did not segregate students based on professional teaching years. More interestingly, antibiotic usage was frequently observed in student belonging to urban areas (82.4%) and those enrolled in bachelor’s degree program (80.9%), the probable reasons could be that students residing in urban areas have an easy access to health education resource, i-e., internet, media, medical education and medicines per se, along with concurrent risk of disregard about the seriousness of a medical condition, respectively–fostering self-medication. Additionally, when stratified for antibiotic classes, b-lactams were frequently and more likely to be used by urban residents, bachelor’s enrollees and un-employed students, while macrolides and quinolones were more frequently used by urban population and pharmacy students, respectively.

Moreover, in developing countries like Pakistan, medicines are sold without even asking for a prescription and pharmacists or even dispensers are treating illness at pharmacies[32,33]. In this regard, the knowledge, preferences, attitudes and experiences of a pharmacist during the course of education and training played a pivotal role in shaping up their appreciation of self-medication and recommendations to peers, friends and community members[34], since pharmacist is central to health care services, mainly offering drug and disease related education, care and counseling[34]. Our data suggested that above 50% started antibiotics without prescription, 60% followed the prescribed dose and yet over 64% did not bother at all in case of a missing dose. However, not a single study from Pakistan is available to compare our data that examined antibiotic use in URIs among pharmacy students, nevertheless, a study from Karachi specifically assessed antibiotic self-medication, irrespective of any infections, in non-medical students and found it to be around 47%with amoxicillin the foremost antibiotic consumed[7], whereas we found augmentin the chief brand consumed by the students. Moreover, majority of the students did not have prior antibiotic experience (> 63%) but preferentially consult doctor in case symptoms persist (> 65%) or illness re-appear (> 51%), whereas non-pharmacy students had higher risks of exposure to antibiotic hazards and re-usage in case of a next attack. Seemingly, university students, irrespective of educational background, are cautious about the symptoms hindering their daily educational performance, but non-pharmacy students having no comprehension of hazards associated with antibiotics and the disease symptoms are likely to experience antibiotic induced adverse effects and consider re-using antibiotics in case of a next attack with similar symptoms.

### Study Limitations

There are a few limitations of the study. The major limitation is the inability to validate the answers by the respondents due to subjective nature of the questionnaire–since respondents might underestimate the actual use of self-medications. Moreover, despite maximizing the efforts to opt randomized sampling, it was not systematic randomization, yet to avoid the convenience effect, we ensured diversity by enrolling students from different universities and by attending all possible student’s gatherings.

### Conclusion

In conclusion, our data suggested that university students, pharmacy and non-pharmacy, are frequent users of self-prescribed antibiotics and drugs to overcome URIs and related symptoms, respectively, probably to warrant un-interrupted study session, to give the best performance, to avoid clinic hassle and to avoid wastage of time. Furthermore, pharmacy education did not promote rational self-prescribing habits, as many think of–evident by the use of antibiotics in common cold. However, pharmacy students were cognizant about the antibiotic hazards and were considering alternative treatment options such as Joshanada, thus seem more cautious. On the other hand, non-pharmacy students were completely oblivious of rational use of antibiotics–even opted to use in pain, and were ready to self-prescribe antibiotics again in case of a next attack.

Therefore, the educational and training programs with frequent crash courses should be designed to counsel the students, irrespective of study course, regarding hazards of self-medication, importance of identifying the medical condition, duration of antibiotic therapy, significance of anti-microbial resistance and to formulate explicit guidelines for the treatment of URIs and associated symptoms frequently encountered by the students. Similarly, clinic visits should be made hassle free that many avoid, and as a result turned towards self-medication.

## Supporting Information

S1 Text Distribution of Non-pharmacy Students, Antibiotic Brands and Side effects. (DOCX)
