# Supplementary material for: Antibiotic Self-Prescribing Trends, Experiences and Attitudes in Upper Respiratory Tract Infection among Pharmacy and Non-Pharmacy Students: A Study from Lahore
Source: PLoS One. 2016 Feb 26;11(2):e0149929. doi: 10.1371/journal.pone.0149929 (PMC4769098; doi:10.1371/journal.pone.0149929)
Supplement: S1 Text — (DOCX) [file pone.0149929.s001.docx]

**Figure A:** Subject Wise Distribution of Non-pharmacy students

**Table A**: Antibiotic Brands Used

| Antibiotics | Pharmacy students  (n=181) | Non pharmacy students (n=199) | Total (n=380) |
| --- | --- | --- | --- |
| augmentin^1^ | 51 (28.17%) | 59 (29.64%) | 110 (28.94%) |
| amoxil^2^ | 43 (23.75%) | 35 (17.58%) | 78 (20.52%) |
| leflox^3^ | 19 (10.49%) | 23 (11.95%) | 42 (11.05%) |
| klaricid^4^ | 18 (9.94%) | 9 (4.52%) | 27 (7.10%) |
| azomax^5^ | 11 (6.07%) | 12 (6.3%) | 23 (6.05%) |
| cefaclor^6^ | 2 (2.17%) | 6 (3.01%) | 8 (2.10%) |
| azithro^7^ | 18 (9.94%) | 5 (2.5%) | 23 (6.05%) |
| novidat^8^ | 12 (6.62%) | 11 (5.52%) | 23 (6.05%) |
| moxiget^9^ | 4 (2.2%) | 3 (1.50%) | 7 (1.84%) |
| velosef^10^ | 4 (2.2%) | 6 (3.01%) | 10 (2.63%) |
| erythro^11^ | 2 (1.10%) | 5 (3.87%) | 7 (1.84%) |
|  | 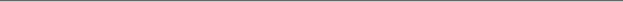 | | |
|  | *^1^Amoxicillin + Clavulonic acid ^2^Amoxicillin ^3^Levofloxacin ^4^Clarithromycin ^5^Azithromycin ^6^Cefaclor ^7^Azithromycin ^8^Ciprofloxacin ^9^Moxifloxacin ^10^Cephradine ^11^Erythromycin* | | |

**Table B**: Antibiotic Use Related Side Effects

| Side effects | Pharmacy students  (n=181) | Non pharmacy students (n=199) | Total (n=380) |
| --- | --- | --- | --- |
| Nausea/vomiting | 30 (16.57%) | 18 (9.04%) | 48 (12.63%) |
| Headache | 47 (25.96%) | 75 (37.68%) | 122 (32.10%) |
| Heartburn | 17 (9.39%) | 15 (7.53%) | 32 (8.42%) |
| Fatigue | 32 (17.67%) | 16 (8.04%) | 48 (12.63%) |
| Abdominal pain | 20 (11.04%) | 16 (8.04%) | 36 (9.47%) |
| Laziness | 51 (28.17%) | 48 (24.12%) | 99 (26.05%) |
| Sleeplessness | 22 (12.15%) | 28 (14.07%) | 50 (13.15%) |
| Palpitation | 12 (6.62%) | 5 (2.51%) | 17 (4.47%) |
